# Supplementary material for: Evidence of Orientation-Dependent Early States of Prion Protein Misfolded Structures from Single Molecule Force Spectroscopy
Source: Biology (Basel). 2022 Sep 16;11(9):1358. doi: 10.3390/biology11091358 (PMC9495685; doi:10.3390/biology11091358)
Supplement: Supplementary file 1 [file biology-11-01358-s001.zip › biology-1876702-supplementary.pdf]

Supplementary Materials

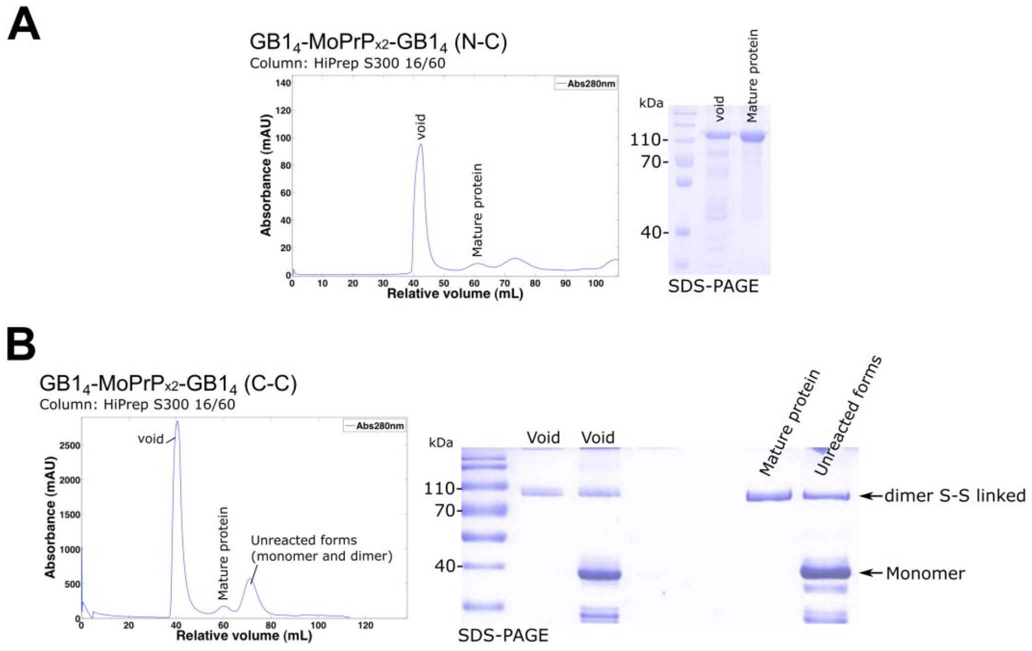

Figure S1. Purification of dimeric PrP constructs used in this study. (**A**) SEC profile at 280 nm showing the elution of $(GB1)_4PrP_2(GB1)_4$ N-C construct (on the left) with the corresponding SDS-PAGE of significant fractions (on the right). (**B**) SEC profile showing the elution of $(GB1)_4PrP_2(GB1)_4$ C-C construct (on the left) with the corresponding SDS-PAGE of significant eluted fractions (on the right). The so-called "mature protein" represents the sample of each dimeric PrP construct used for the AFM-SMFS experiments. Notably, in the SDS-PAGE of panel (**B**) a remaining "mature protein" is present also in the fraction corresponding to the peak called "Unreacted forms": we believe that this dimer derives from an incomplete peak separation, while the main peak contribution is given by the monomeric form, which is the mostly represented specie.

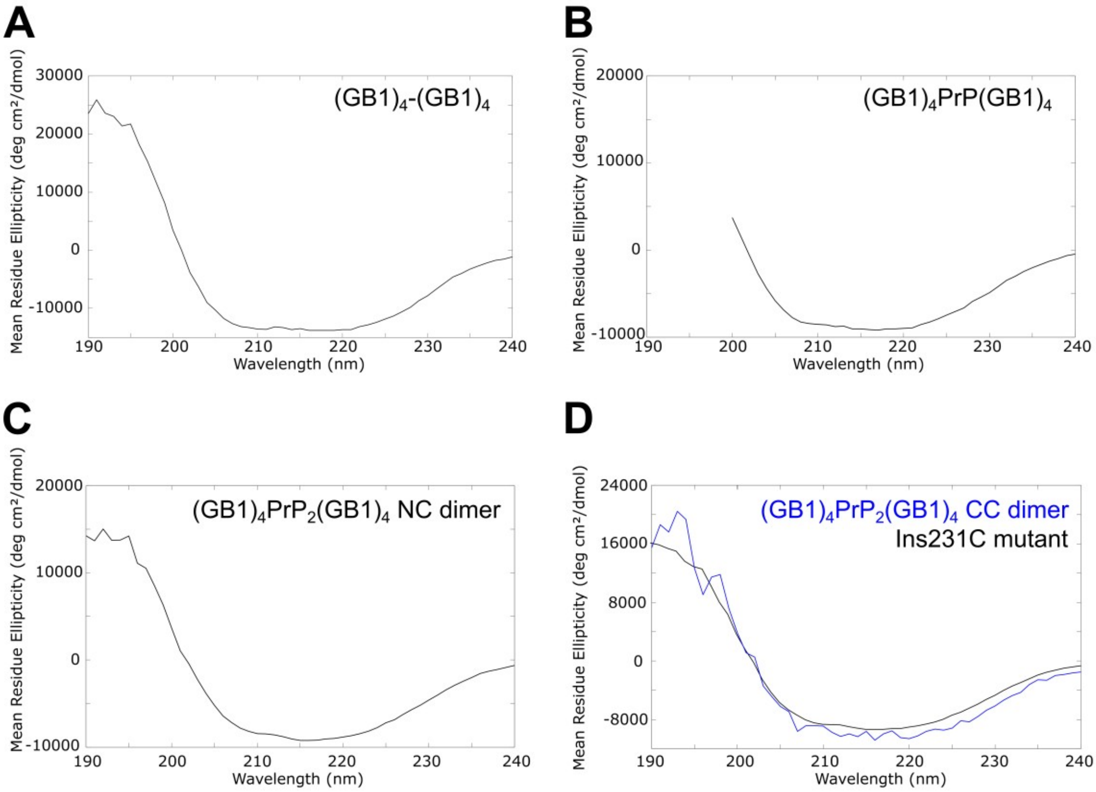

Figure S2. Circular dichroism (CD) spectra of hetero-polymeric constructs used in this study. In (**A**) the spectrum of the $(GB1)_4$-$(GB1)_4$ construct, while in (**B**) of monomeric PrP flanked by 4 GB1 proteins at both N- and C-termini, $(GB1)_4PrP(GB1)_4$. This spectrum has been cut at 200 nm due to high scattering signals in the 190-200 nm region. In (**C**) and (**D**), the spectra of dimeric constructs: $(GB1)_4PrP_2(GB1)_4$ N-C and $(GB1)_4PrP_2(GB1)_4$ C-C, respectively. All spectra have been normalized on protein residues concentration. The spectra show two characteristic minima at around 208 and 222 nm that confirm the presence of both α-helical and β-sheet secondary structures in the folding of the protein constructs used in this study. The spectrum corresponding to the C-C dimer has a lower mean residue ellipticity and high noise-ratio due to the very low concentration of the purified protein sample; despite this the spectrum suggests the also this construct is folded. In (**D**) we also loaded the spectrum of the Ins231C mutant, i.e., the $(GB1)_4$PrP-Cys231 prior to the dimerization with itself, showing identical profile as the CC dimer and suggesting that no major conformational changes occur after the linkage.

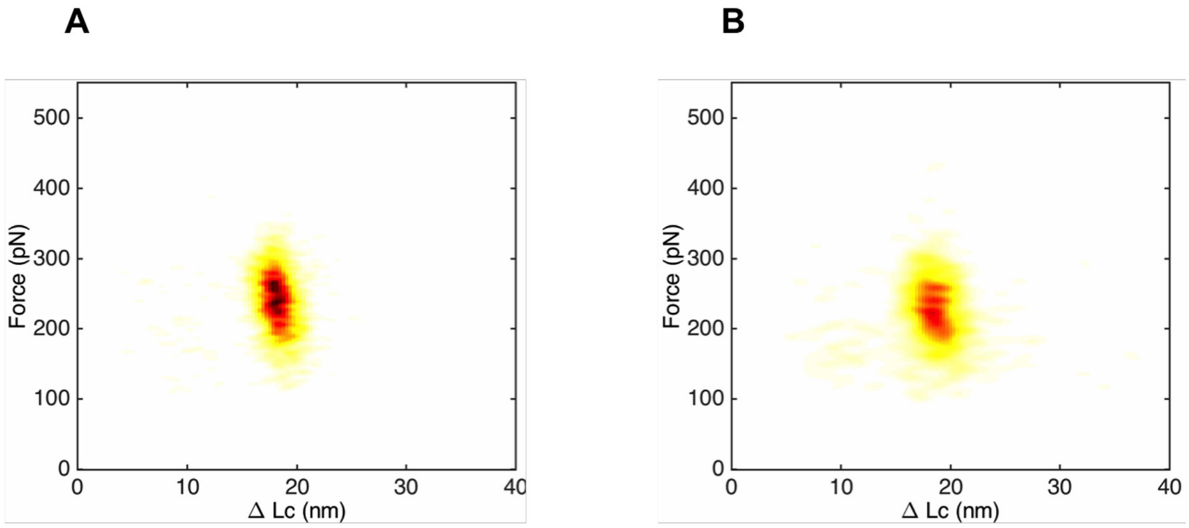

Figure S3. Kernel density estimation distribution obtained from all unfolding events of $(GB1)_4$-$(GB1)_4$ reference construct (**A**) and monomeric $(GB1)_4$PrP$(GB1)_4$ construct (**B**). $(GB1)_4$-$(GB1)_4$ unfolding events are clustered at 18.5 ± 0.9 nm and 240 ± 40 pN at this force-loading rate. Longer unfolding events are more frequent in monomeric $(GB1)_4$PrP$(GB1)_4$ construct but are not clearly visible in this representation that privileges the more probable unfolding events.

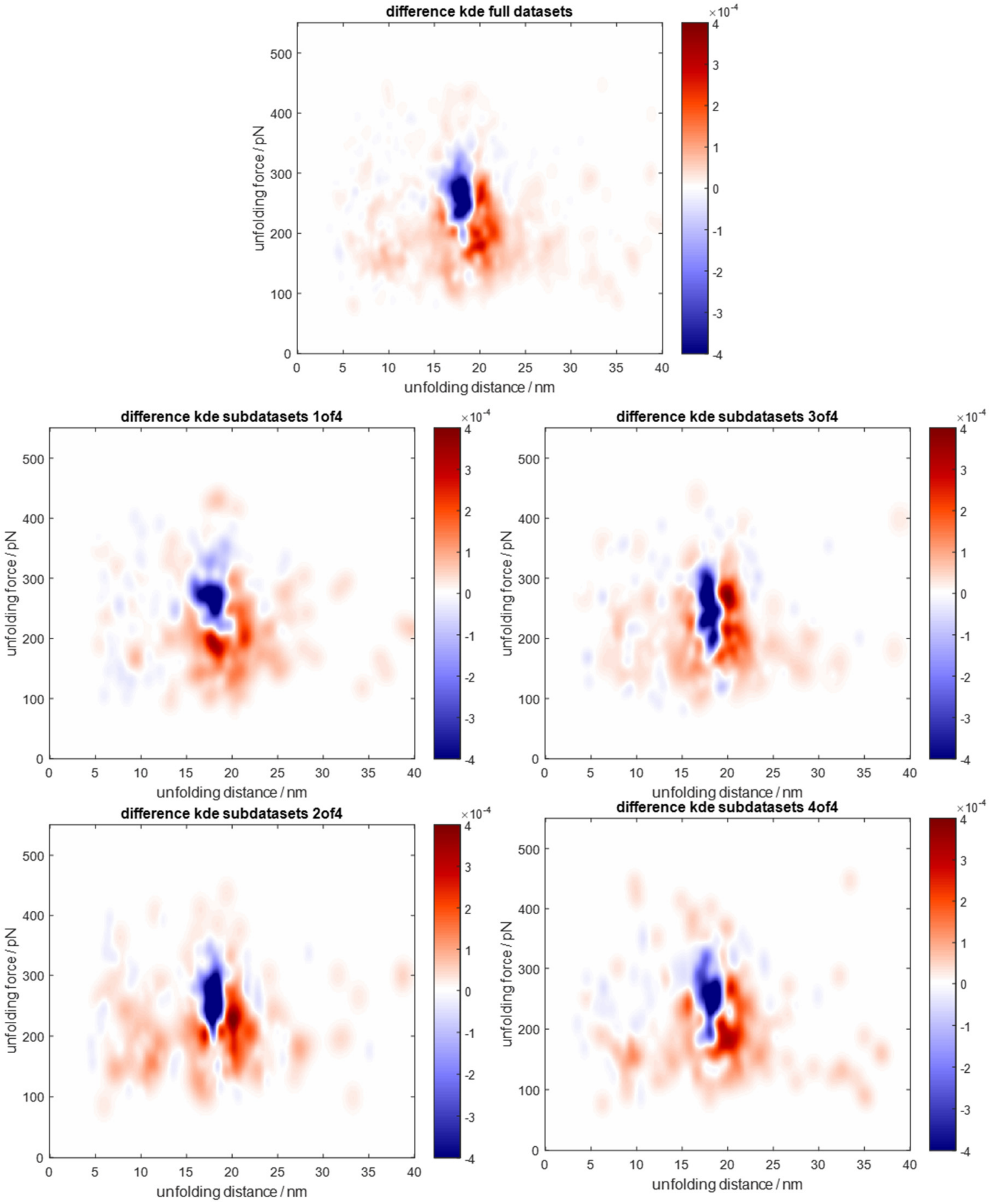

Figure S4: Comparison between the differential kernel density estimation probability heat map of the unfolding events between the (GB1)$_4$PrP(GB1)$_4$ construct and the (GB1)$_4$-(GB1)$_4$ reference construct at neutral pH. The plots are calculated on the entire dataset (top) and on 4 randomly chosen (non overlapping) subsets. The analysis shows that, albeit with fluctuations in shape, the evidence on the SMFS signal of the native fold of PrP is statistically significant in the reported data (here evident as the red-colored area in the region at around 200 pN and approximately 20-22 nm).

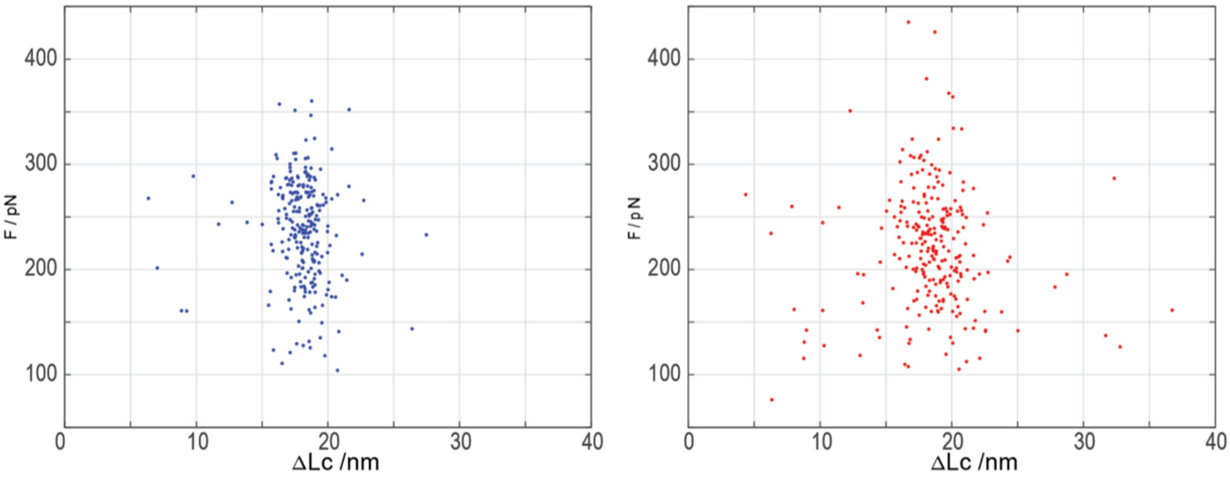

Figure S5. Scatter plots of subsets of the same size of the unfolding events recorded for the pulling of molecular constructs of $(GB1)_4$-$(GB1)_4$, on the left, and $(GB1)_4PrP(GB1)_4$, on the right.

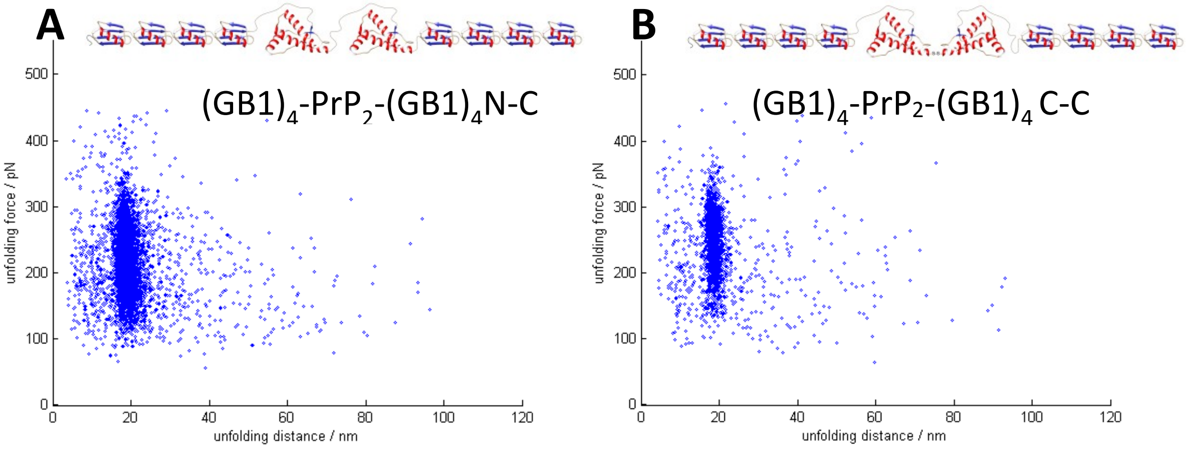

Figure S6. Full available datasets for the scatter plots of force unfoldings of the PrP dimers: on panel **A**, the N-C linked dimers; on panel **B**, the C-C linked dimers.

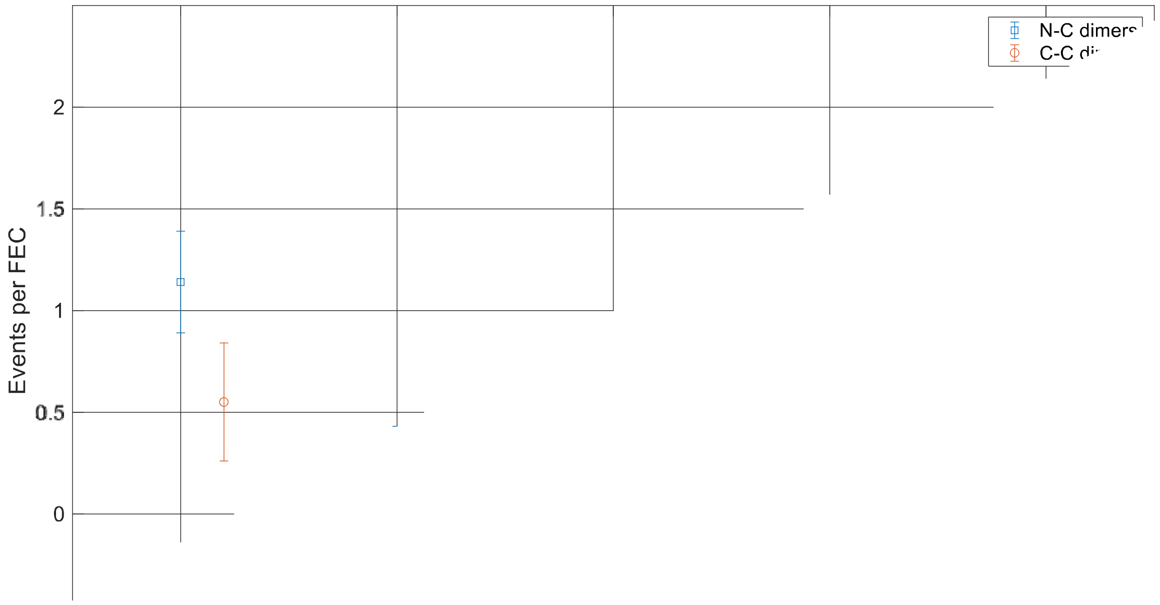

Figure S7. Relative conformer frequencies (unfolding events per force-extension curve in the named ΔLc region) of dimeric N-C (blue) and dimeric C-C constructs (black). Unfolding of the C-terminal structured domain of PrP is marked as "native": for N-C it was calculated in the region as defined from the monomeric native constructs of PrP (21.5 ± 2.5 nm and 210 ± 70 pN) while for the C-C dimer it was computed for a shifted region (19.1 ± 2.0 nm, 290 ± 40 pN) as evident from inspection of the differential KDE plots (see Fig. 4 of the main text). Error bars are ± the weighted standard deviation of the events per curve as calculated for the means obtained for each experiment performed with a sample, weighted for the number of pulled molecule in theexperiment.

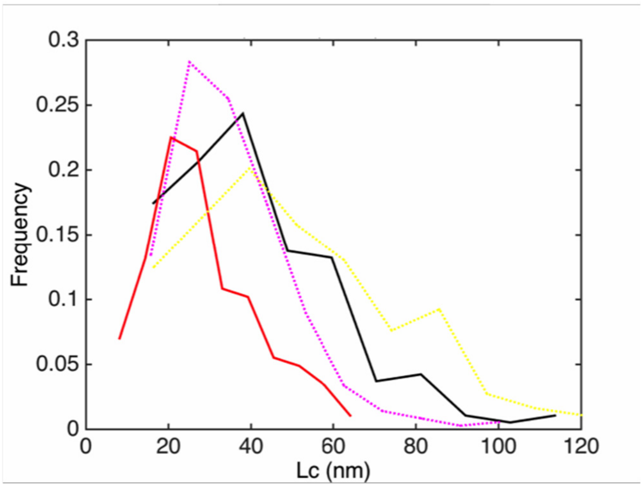

Figure S8. Distribution of the contour lengths of the first peaks in the unfoldings. $(GB1)_4$-$(GB1)_4$ in red, monomeric $(GB1)_4PrP(GB1)_4$ in black, monomeric $(GB1)_4C_{term}$-$PrP(GB1)_4$ in magenta and reduced monomeric $(GB1)_4PrP(GB1)_4$ in yellow.
